# Accuracy, Reliability, and Quality of YouTube and TikTok Videos as Sources of Information on Urticaria: Cross-Sectional Study

**DOI:** 10.2196/88827

**Published:** 2026-11-09

**Authors:** Abrar Bukhari, Saad Altalhab, Suliman AlFaraj, Huda Alrwebah, Mishari Alrubaiaan, Abdulrahman Alfawzan

**Affiliations:** 1 Department of Dermatology, College of Medicine, Imam Mohammad Ibn Saud Islamic University (IMSIU), Riyadh, Saudi Arabia; 2 Department of Dermatology and Dermatologic Surgery, Prince Sultan Military Medical City, Riyadh, Saudi Arabia; 3 Department of Pediatric Dermatology, King Abdullah Specialized Children’s Hospital, National Guard Health Affairs (NGHA), Riyadh, Saudi Arabia; 4 Department of Dermatology, King Abdulaziz Medical City, National Guard Health Affairs (NGHA), King Abdullah District, 4551, Riyadh, 12444, Saudi Arabia

**Keywords:** urticaria, social media, YouTube, TikTok, DISCERN, Global Quality Score, GQS, Accuracy in Digital-Health Instrument, ANDI, content quality, health care professionals, patient uploaders

## Abstract

**Background:**

The era of social media platforms has transformed the dissemination of health information, raising concerns about the quality and accuracy of content. Urticaria, a common skin condition, necessitates reliable information for effective management. Unreliable information on the internet can lead to misconceptions about urticaria, which could contribute to overemphasis on triggers or underrepresentation of guideline-based interventions, delaying effective management approaches.

**Objective:**

This study aimed to systematically evaluate urticaria-related content on 2 prominent social media platforms, YouTube and TikTok, using validated tools to assess information reliability and explore the characteristics of videos and their uploaders.

**Methods:**

On February 16, 2023, a comprehensive search on YouTube and TikTok was conducted for videos related to urticaria. Two independent dermatologists rated 92 eligible videos using DISCERN, the Global Quality Score (GQS), and the Accuracy in Digital-Health Instrument (ANDI). Statistical analyses, including mean values, SDs, percentages, and chi-square tests, were used to interpret the data.

**Results:**

TikTok videos were predominantly uploaded by patients (29/46, 63%), whereas YouTube exhibited a higher engagement of health care professionals (31/46, 67.4%). YouTube videos exhibited higher mean scores for the GQS (2.9, SD 0.7), ANDI (2.3, SD 0.8), and DISCERN (40.2, SD 7.6) compared to TikTok (GQS: mean 2, SD 0.8; ANDI: mean 1.4, SD 1; DISCERN: mean 32.9, SD 7.9). Videos uploaded by professionals on TikTok had significantly higher mean GQS (2.8, SD 0.7), ANDI (2.1, SD 0.8), and DISCERN (40, SD 6.3) scores compared to those uploaded by nonprofessionals (*P*=.001). Similarly, videos from professional uploaders on YouTube exhibited significantly higher mean GQS (3, SD 0.6), ANDI (2.6, SD 0.6), and DISCERN (42.2, SD 7.1) scores compared to nonprofessionals (*P*=.01 for GQS, *P*=.001 for ANDI, and *P*=.003 for DISCERN).

**Conclusions:**

Urticaria videos on TikTok showed a significantly higher engagement ratio and less accuracy and quality of health-related information than YouTube videos. These insights can guide content creators, platform administrators, and health care professionals in collaboratively fostering a trustworthy and accurate health information environment on social media.

## Introduction

Urticaria, commonly known as hives, is a prevalent dermatological condition characterized by the sudden appearance of red, itchy edematous skin lesions. The condition has a lifetime prevalence of nearly 20% [1]. These lesions vary in size and shape and often emerge as a result of an allergic reaction or other triggers, such as immunoglobulin E (IgE)–mediated hypersensitivity to drugs or insect venoms and non–IgE-mediated mechanisms, that prompt the release of histamine from mast cells [2-4]. Non–IgE-mediated mechanisms in chronic spontaneous urticaria are triggered by immune complexes that bind immunoglobulin M and immunoglobulin G [5,6], whereas chronic inducible urticaria is triggered by physical stimuli, vaccines, or infections (viruses, parasites, and bacteria) [2]. The condition’s transient nature and the potential for severe discomfort make it a significant health concern affecting individuals of all ages [7]. Urticaria management is based on established clinical recommendations, which require medical practitioners to follow the clinical history (to make the initial diagnosis and examine the etiological aspects involved), carry out diagnoses to rule out other dermatological disorders, and examine the skin to detect the lesions [1].

Following examination, medical practitioners can recommend a standard dose of second-generation H1 receptor antihistamines as the first-line treatment [8]. Among individuals with persistent symptoms, the guidelines recommend increasing the dose of second-generation antihistamines up to four-fold as the second-line treatment [9,10]. In refractory cases, biologic treatment can be used to attain spontaneous resolution [11]. According to the American Academy of Allergy, Asthma & Immunology, there are various misconceptions concerning urticaria etiology, especially over attribution to dietary, stress, or pet causes [12]. Such misconceptions mostly contribute to patients seeking alternative interventions and explanations through digital platforms, smart devices, and computers outside formal medical guidance [13]. Additionally, while medical interventions are available for managing urticaria, the accessibility of information through digital platforms has led many individuals to seek insights and guidance from online sources [14].

The advent of social media has transformed the landscape of health communication, providing a platform for individuals to share personal experiences, seek advice, and access information [15]. User engagement in social media platforms has witnessed a substantial rise over the past years. This is evidenced by the fact that over half of the population in the world has an online social media account [16]. Such a rise was further intensified due to the global COVID-19 pandemic [17]. YouTube and TikTok, among other applications, have repeatedly proven to be the platforms with the most traffic from online consumers [18]. With millions of health-related videos circulating on these platforms and their broad reach and impactful potential [19,20], social media can act as a catalyst to propel valid and reliable educational materials forward [20].

The democratization of content creation allows patients, health care professionals, and various entities apart from health care organizations to contribute to the discourse surrounding health conditions. However, the unregulated nature of these platforms raises questions about the accuracy and quality of health-related information, particularly concerning dermatological conditions such as urticaria [21]. Generally, social media ranking algorithms optimized for users have revealed preferences, including user engagement such as comments, shares, likes, and watch times, to determine which content is ranked or pushed to wider audiences [22]. By concentrating on user engagement, the algorithms might amplify content that is popular but unaligned with scientific information or evidence-based guidelines. Consequently, low-quality or misleading medical-related content might gain traction to wider coverage, potentially shaping public perceptions, impacting decisions, and amplifying misinformation [23]. Such concerns are compounded by social media’s widespread amplification of unverified information, which underscores the significance of systematically evaluating the reliability, quality, and accuracy of health-related content available on such platforms.

Health information on social media is often characterized by its dynamic and diverse nature. Patients share personal anecdotes, health care professionals offer expert advice, and entities ranging from medical groups to news companies contribute to the content pool [15]. Understanding the characteristics of urticaria-related content on platforms such as YouTube and TikTok is crucial considering the potential impact on public knowledge, perceptions, and health care–seeking behaviors [24].

The influence of social media on health-related decisions is a complex interplay of factors [19,25]. Patients often turn to these platforms seeking information that complements or even substitutes traditional health care channels [25]. The allure of relatable stories and visual content can provide a sense of connection and understanding, fostering a community of individuals navigating similar health challenges [24,25]. However, the lack of regulation and oversight raises concerns about the accuracy and reliability of the information disseminated, especially when it comes to conditions such as urticaria, where misinformation can impact treatment choices and overall well-being [21,26,27].

The concept of health literacy in the digital age has garnered attention, emphasizing individuals’ ability to access, understand, and apply health information from online sources [24,28]. For dermatological conditions such as urticaria, where self-management and lifestyle choices play a role, the impact of online information becomes even more pronounced. As individuals navigate the plethora of content available, it becomes imperative to assess the quality, accuracy, and reliability of health-related information on these platforms [27].

Existing literature highlights the challenges posed by health misinformation on social media and its potential consequences for public health [25]. While studies have explored the landscape of health-related content on platforms such as YouTube, TikTok’s role in shaping health perceptions is relatively uncharted territory. TikTok’s format, characterized by short videos and a younger user demographic, introduces unique considerations in understanding the dynamics of health communication [29,30]. Nevertheless, the lack of content regulation on social media platforms raises concerns about the reliability and accuracy of the information disseminated. In the context of urticaria, misinformation might promote misconceptions on the etiology (eg, diet, treatments, causes, and supplementation), potentially contributing to delayed management [31].

As the prevalence of urticaria persists and individuals increasingly turn to online platforms for health information, there is a pressing need to critically examine the quality of content available. This study endeavored to fill a gap in the literature by systematically evaluating urticaria-related content on YouTube and TikTok. We aimed to assess and compare the quality, accuracy, and reliability of urticaria-related information available on YouTube and TikTok using validated assessment tools. Given the variations in the presentation, content creation features, and format of the videos between the 2 platforms, we hypothesized that the reliability and accuracy of the information would vary. Through the application of validated scoring criteria and a rigorous methodology, we aimed to provide insights into the characteristics of videos, the role of different uploader types, and the overall quality of the health information available on these platforms. The findings of this study hold implications for health care professionals, content creators, and platform administrators in shaping a reliable and informative health communication landscape in the digital age.

## Methods

### Study Design

This study used a cross-sectional observational design to assess the quality of urticaria-related content on YouTube and TikTok.

### Search Strategy

#### Search Terms

The search for relevant content was conducted on February 16, 2023. To ensure a comprehensive representation of urticaria-related videos, we used clinically and standardized search terms: “urticaria” and “hives.”

#### Search Procedure and Sampling Strategy

On TikTok, searches were performed in both incognito mode and through an author’s account. The results from both approaches were merged before assessing duplication and screening according to eligibility criteria. The first or top 50 videos for each search term were selected, sorted by views, and included in the dataset to ensure relevant sorting. Duplicate videos were defined as identical videos uploaded several times (including by different users). We only relied on unique videos (1 instance) to ensure that observation of each one represented unique content while avoiding overrepresentation of duplicated material. We retained only 1 version of the content when it was uploaded multiple times. We identified duplicates through comparison of the upload metadata, audiovisual content, titles, and platform-specific video identifiers (URLs).

A parallel process was applied to YouTube. Duplicate videos were removed to ensure the uniqueness of the dataset.

#### Eligibility Criteria

We included publicly accessible English-language videos directly related to urticaria and excluded videos exceeding 20 minutes in duration.

### Video Classification and Characteristics

The final dataset consisted of 92 videos, reflecting a diverse representation of urticaria-related content. The compiled dataset underwent thorough characterization, extracting key variables for analysis. Video characteristics that we included were views; likes; shares; followers; verification status; gender; and presence of animation, subtitles, music, written material, educational content, anecdotal information, home remedies, advertisements, and other elements. We categorized the videos based on the uploader type, specifically, health care professionals or nonprofessionals. Furthermore, we classified video content types as animation, music, educational, anecdotal, home remedy, advertisement, written material, and subtitles.

### Quality Assessment

Two independent expert dermatologists were involved in the video assessment process, ensuring a comprehensive and professional assessment. To evaluate the quality and accuracy of urticaria-related content, 3 established tools were used: DISCERN [32], the Global Quality Score (GQS) [33], and the Accuracy in Digital-Health Instrument (ANDI) [34]. DISCERN is a tool comprising 16 questions rated from 1 to 5, where 1 means “no” and 5 means “yes,” while 3 is “partial” [32]. It measures 3 aspects, including reliability, quality of information, and overall rating of the videos. The GQS and ANDI’s primary aim is to provide a standardized method for assessing the overall quality of digital health content. All tools used are provided in Multimedia Appendix 1.

### Platform Comparisons

Comparisons were performed between TikTok and YouTube to assess differences in video characteristics and quality assessment scores. Within each platform, videos were also compared according to uploader type (professional vs nonprofessional) with respect to views; likes; shares; followers; daily views; engagement percentage; video duration; and DISCERN, GQS, and ANDI scores. DISCERN score categories were also compared between professional and nonprofessional uploaders within each platform.

### Statistical Analysis

We summarized video characteristics and selection using means and SDs provided they were continuous variables, while we used frequencies and percentages for categorical variables. Similarly, we used means and SDs to summarize quality assessment of TikTok and YouTube urticaria videos and platform comparison. The distribution of continuous variables was assessed using the Shapiro-Wilk test. Normally distributed continuous variables were summarized as means and SDs and compared using parametric tests, whereas nonnormally distributed variables were summarized as medians and IQRs and compared using nonparametric tests. For comparisons between TikTok and YouTube, the 2-tailed independent-samples *t* test was used for normally distributed continuous variables, and the Mann-Whitney *U* test was used for nonnormally distributed continuous variables. Categorical variables were compared using the chi-square test. The level of significance was set at .05.

## Results

### Search Results

On TikTok, 63 videos were identified through the incognito search, and 76 were identified through the author’s account. Across the 2 search terms, “urticaria” retrieved 75 videos, whereas “hives” retrieved 64 videos. Following the removal of duplicates and irrelevant videos based on the inclusion criteria, 46 unique TikTok videos were included in the final analysis. On YouTube, the search yielded 85 videos, of which 51 were retrieved using “urticaria” and 34 were retrieved using “hives.” Following deduplication and application of the inclusion criteria, 46 unique YouTube videos were included in the final analysis. Overall, 92 videos, comprising 46 videos from each platform, were included in the final analysis.

### Video Classification and Characteristics

Table 1 shows the characteristics of the uploading channels and uploaded videos on TikTok and YouTube, providing insights into the diverse landscape of content creators and their respective platforms. All TikTok videos had humans, while 80.4% (37/46) of the videos on YouTube had humans. In addition, there was a predominant presence of patients on TikTok (29/46, 63%) compared to YouTube (5/46, 10.9%).

**Table 1.** Characteristics of uploading channels and uploaded videos on TikTok and YouTube (N=46).

Parameters	TikTok	YouTube
Uploader, n (%)		
Not professional	31 (67.4)	15 (32.6)
Professional	15 (32.6)	31 (67.4)
Views		
Mean (SD; range)	631,513 (1,040,097; 63,300-4,800,000)	171,130 (336,435; 11,000-1,800,000)
Median (IQR)	330,050 (102,625-526,150)	62,000 (35,000-179,000)
Likes		
Mean (SD; range)	29,688 (66,353; 175-324,200)	1761 (3046; 0-17,000)
Median (IQR)	3907 (621-26,050)	595 (308-1425)
Shares		
Mean (SD; range)	410 (772; 11-4259)	419 (614; 0-2916)
Median (IQR)	104 (40-407)	189 (50-529)
Verified, n (%)	3 (6.5)	21 (45.7)
Sex, n (%)		
No human in the video	0 (0)	9 (19.6)
Male	8 (17.4)	13 (28.3)
Female	38 (82.6)	18 (39.1)
Both	0 (0)	6 (13)
Animation, n (%)	1 (2.2)	11 (23.9)
Subtitles, n (%)	11 (23.9)	6 (13)
Music, n (%)	26 (56.5)	23 (50)
Written material, n (%)	44 (95.7)	42 (91.3)
Educational, n (%)	19 (41.3)	39 (84.8)
Anecdotal, n (%)	27 (58.7)	9 (19.6)
Home remedy, n (%)	4 (8.7)	13 (28.3)
Advertisement, n (%)	3 (6.5)	12 (26.1)

Engagement metrics varied across the 2 platforms, as evidenced by the differences in the mean views, likes, shares, and followers between TikTok and YouTube. TikTok videos exhibited a higher mean number of views (631,513, SD 1,040,097; median 330,050, IQR 102,625-526,150) compared to YouTube (mean 171,130, SD 336,435; median 62,000, IQR 35,000-179,000). Additionally, TikTok videos garnered substantially more likes (mean 29,688, SD 66,353; median 3907, IQR 621-26,050) and shares (mean 410, SD 772; median 104, IQR 40-407) than YouTube videos (mean 1761, SD 3046 and median 595, IQR 308-1425 likes; mean 419, SD 614 and median 189, IQR 50-529 shares). In addition, YouTube exhibited slightly comparable mean numbers of shares (419, SD 614) to those of TikTok (410, SD 772). Moreover, a noteworthy proportion of YouTube videos were verified (21/46, 45.7%), indicating potential credibility, while most of the videos on TikTok (43/46, 93.5%) were not.

TikTok videos were less likely to include animation (1/46, 2.2%) and more likely to include subtitles (11/46, 23.9%) compared to YouTube (11/46, 23.9% and 6/46, 13%, respectively). Health professionals, including physicians, nonphysicians, and medical groups or foundations, were more active on YouTube (31/46, 67.4%) than on TikTok (15/46, 32.6%). Written material content comprised most videos across TikTok (44/46, 95.7%) and YouTube (42/46, 91.3%), followed by educational and musical content.

### Quality Assessment

Regarding the evaluation of videos using DISCERN, GQS, and ANDI scores by 2 independent expert dermatologists, the DISCERN scores, indicative of the quality of information, demonstrated a moderate level, with rater 1 assigning a mean score of 37 (SD 11) and rater 2 assigning a mean score of 36 (SD 13). The interrater reliability, as measured using the Cronbach α, was low (0.102), suggesting variations in scoring between the raters. The GQS and ANDI scores exhibited higher levels of agreement between raters, with mean scores of 3 (SD 1) and 3 (SD 1), respectively, and higher interclass correlation coefficients (0.619 and 0.766, respectively).

Table 2 delves into the comparison of average DISCERN, GQS, and ANDI scores across different platforms. Notably, YouTube videos demonstrated higher mean scores for the GQS (2.9, SD 0.7), ANDI (2.3, SD 0.8), and DISCERN (40.2, SD 7.6) compared to TikTok (GQS: mean 2, SD 0.8; ANDI: mean 1.4, SD 1; DISCERN: mean 32.9, SD 7.9), indicating a potential disparity in the quality and accuracy of the information between the 2 social media platforms (*P*=.001).

**Table 2.** Average DISCERN, Global Quality Score (GQS), and Accuracy in Digital-Health Instrument (ANDI) scores among different platforms.

Items	Platform, mean (SD)		Mean difference (95% CI)	*P* value^a^
	TikTok	YouTube		
GQS (1-5)	2 (0.8)	2.9 (0.7)	−0.82 (–1.5 to –0.59)	.001
ANDI (0-4)	1.4 (1)	2.3 (0.8)	−0.95 (–1.5 to –0.59)	.001
DISCERN (16-80)	32.9 (7.9)	40.2 (7.6)	−7.28 (–1.4 to –0.51)	.001

a The 2-tailed independent-samples *t* test was used to calculate the *P* values.

### Platform Comparisons

Table 3 provides a comprehensive analysis of TikTok videos, categorizing them based on the uploader type and comparing various metrics such as views, likes, shares, followers, daily views, engagement percentage, and video duration. Interestingly, TikTok videos from nonprofessionals exhibited higher mean views (670,129, SD 981,086) than those from professionals (551,707, SD 1,185,129), although the difference was not statistically significant (*P*=.72). However, nonprofessionals had nonsignificantly higher shares (mean 397, SD 778) compared to professionals (mean 437, SD 786; *P*=.87). The daily views (*P*=.84) and engagement percentages (*P*=.08) did not show significant differences between the two 2 groups. In terms of video quality assessment, videos from professionals on TikTok had significantly higher mean GQS (2.8, SD 0.7), ANDI (2.1, SD 0.8), and DISCERN (40, SD 6.3) scores compared to nonprofessionals (*P*=.001 for all three comparisons), indicating a potential association between professional status and the quality of urticaria-related content on TikTok (Figure 1).

**Table 3.** Video characteristics and average DISCERN, Global Quality Score (GQS), and Accuracy in Digital-Health Instrument (ANDI) scores among TikTok videos compared by uploader type.

Items	Nonprofessionals	Professionals	*P* value^a^	Mean difference (95% CI)
Views			.72	—^b^
Mean (SD)	670,129 (981,086)	551,707 (1,185,129)		
Median (IQR)	61,000 (43,000-171,000)	69,000 (34,500-195,500)		
Likes			.60	—
Mean (SD)	33,337 (64,594)	22,145 (71,556)		
Median (IQR)	552 (292-1900)	631 (321-1450)		
Shares			.87	—
Mean (SD)	397 (778)	437 (786)		
Median (IQR)	196 (24-667)	181 (61-485)		
Followers			.09	—
Mean (SD)	87,335 (151,167)	891,109 (2,598,803)		
Median (IQR)	189,000 (6120-1,532,500)	148,000 (22,800-1,655,000)		
Daily views, mean (SD)	2798.1 (4318)	3089.9 (4644.1)	.84	—
Engagement (%), mean (SD)	5.84 (7.73)	2.15 (2.68)	.08	—
Duration (s), mean (SD)	46 (45)	58 (46)	.44	—
GQS (1-5), mean (SD)	1.7 (0.7)	2.8 (0.7)	.001	0.61 (0.20-1.53)
ANDI (0-4), mean (SD)	1 (0.9)	2.1 (0.8)	.001	1.01 (0.76-2.19)
DISCERN (16-80), mean (SD)	29.5 (6.2)	40 (6.3)	.001	7.14 (0.35-1.70)

a *P* values are unadjusted. The 2-tailed independent-samples *t* test was used to calculate them.

b Not applicable.

**Figure 1.**
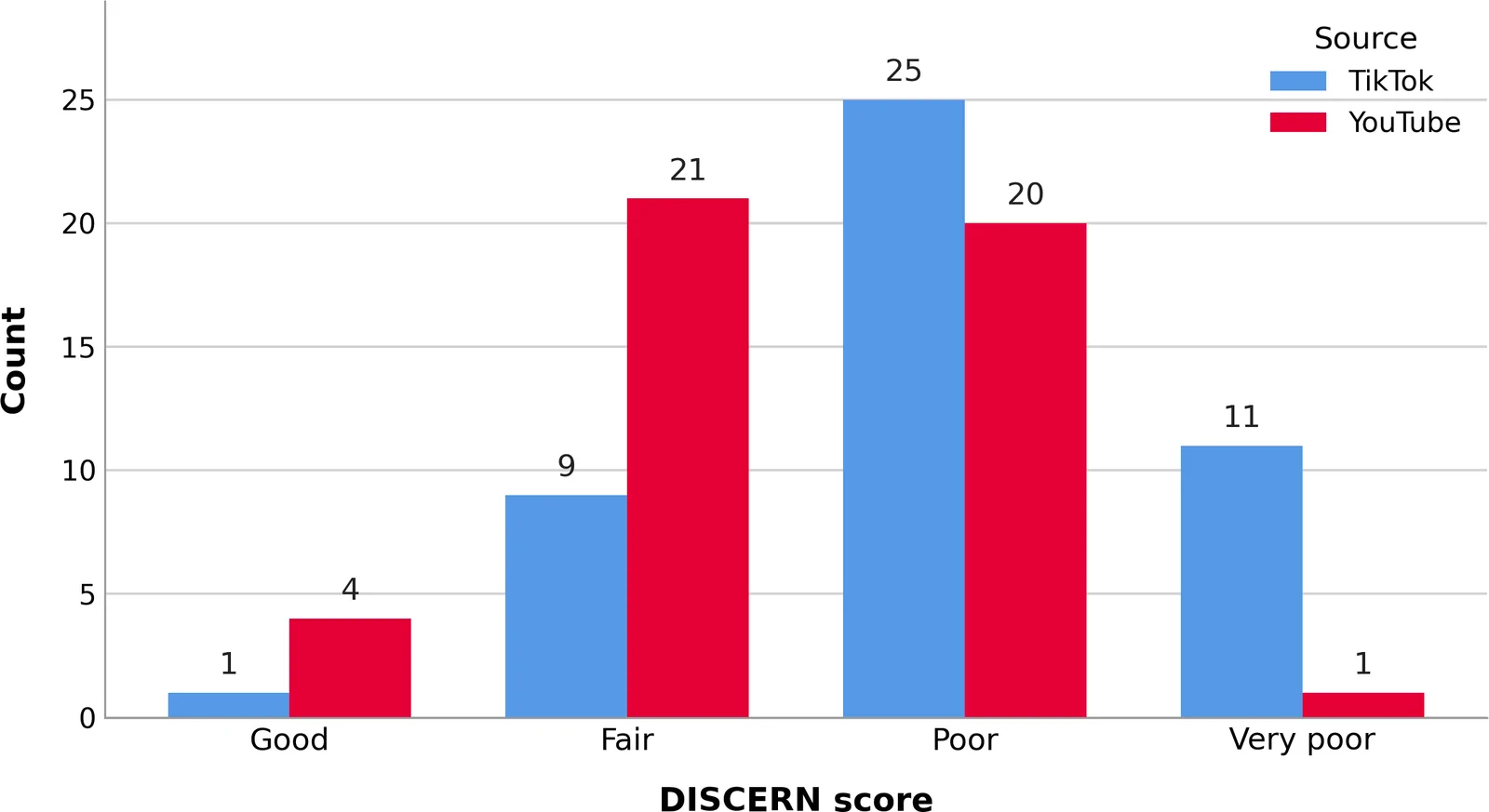
DISCERN score categories across different platforms.

Table 4 extends a similar comparison to YouTube videos based on uploader types. Unlike on TikTok, YouTube videos from professionals had higher mean views (194,545, SD 391,001) compared to nonprofessionals (111,692, SD 106,941), although the difference was not statistically significant (*P*=.46). No significant differences were observed in likes (*P*=.56), shares (*P*=.99), followers (*P*=.41), daily views (*P*=.40), engagement percentage (P=.61), and video duration (P=.30) between nonprofessional and professional YouTube uploaders. However, videos from professional uploaders on YouTube exhibited significantly higher mean GQS (3, SD 0.6), ANDI (2.6, SD 0.6), and DISCERN (42.2, SD 7.1) scores compared to nonprofessionals (*P*=.01 for GQS, *P*=.001 for ANDI, and *P*=.003 for DISCERN), suggesting a potential association between professional status and the quality of urticaria-related content on YouTube.

**Table 4.** Video characteristics and average DISCERN, Global Quality Score (GQS), and Accuracy in Digital-Health Instrument (ANDI) scores among YouTube videos compared by uploader type.

Items	Nonprofessionals	Professionals	*P* value^a^	Mean difference (95% CI)
Views			.46	—^b^
Mean (SD)	111,692 (106,941)	194,545 (391,001)		
Median (IQR)	375,700 (83,300‐757,600)	237,200 (134,300‐368,200)		
Likes			.56	—
Mean (SD)	1342 (1480)	1927 (3482)		
Median (IQR)	5389 (784‐46,900)	138 (331-7659)		
Shares			.99	—
Mean (SD)	421 (625)	418 (619)		
Median (IQR)	149 (30-498)	74 (43-298)		
Followers			.41	—
Mean (SD)	1,641,722 (4,006,427)	930,805 (1,834,514)		
Median (IQR)	14,100 (2894‐78,000)	61,700 (23,400‐547,500)		
Daily views, mean (SD)	101.5 (162.3)	190.1 (355.6)	.40	—
Engagement (%), mean (SD)	1.21 (0.54)	1.1 (0.67)	.61	—
Duration (s), mean (SD)	401 (325)	305 (259)	.30	—
GQS (1-5), mean (SD)	2.4 (0.9)	3 (0.6)	.01	1.07 (0.86-2.25)
ANDI (0-4), mean (SD)	1.6 (0.9)	2.6 (0.6)	.001	1.10 (0.58-1.92)
DISCERN (16-80), mean (SD)	35.1 (6.6)	42.2 (7.1)	.003	8.16 (0.98-2.41)

a The 2-tailed independent-samples *t* test was used to calculate the *P* values.

b Not applicable.

Table 5 delves into the distribution of DISCERN score categories among professional and nonprofessional uploaders on TikTok and YouTube. On TikTok, among videos categorized as “fair,” 77.8% (7/9) were uploaded by professionals. Among videos categorized as “poor,” 72% (18/25) were uploaded by nonprofessionals, while all videos categorized as “very poor” (11/11, 100%) were uploaded by nonprofessionals. This discrepancy was statistically significant (*χ*^2_3_^ =16.0; *P*=.001). On YouTube, the distribution of DISCERN score categories did not reach statistical significance (*χ*^2_3_^=6.4; *P*=.10), although videos from professionals tended to fall into the higher categories (“good” and “fair”) compared to nonprofessionals.

**Table 5.** DISCERN score categories among professional vs nonprofessional uploaders on TikTok and YouTube.

DISCERN score (16-80)	Uploaders, n/N (%)		Chi-square (*df*)	*P* value^a^
	Nonprofessional	Professional		
TikTok			16.0 (3)	.001
Very good	0 (0)	0 (0)		
Good	0 (0)	1/1 (100)		
Fair	2/9 (22.2)	7/9 (77.8)		
Poor	18/25 (72)	7/25 (28)		
Very poor	11/11 (100)	0 (0)		
YouTube			6.4 (3)	.10
Very good	0 (0)	0 (0)		
Good	0 (0)	4/4 (100)		
Fair	4/21 (19)	17/21 (81)		
Poor	8/20 (40)	12/20 (60)		
Very poor	1/1 (100)	0 (0)		

a The chi-square test was used to calculate the *P* values.

Figure 1 shows the DISCERN categories among TikTok and YouTube videos. Under the “good” and “fair” categories, YouTube videos were more predominant than TikTok videos. On the other hand, TikTok videos were predominant under the “poor” and “very poor” categories compared to YouTube videos.

## Discussion

### Video Characteristics

Urticaria, characterized by the sudden onset of raised, itchy welts on the skin, poses a significant burden on individuals and necessitates reliable information sources for effective management [4]. With the rise of the social media era, platforms such as YouTube and TikTok have become accessible venues for disseminating health-related content [24]. However, the quality and accuracy of health information on these platforms remain a concern. In this context, our study aimed to systematically evaluate urticaria-related content on YouTube and TikTok using validated tools to assess the information’s reliability and the characteristics of the videos and their uploaders.

Our comprehensive analysis of 92 videos, spanning diverse characteristics and platforms, revealed intriguing patterns in content quality and uploader types. TikTok videos had almost 4 times the views of YouTube videos and more than 20 times the daily views, with a much higher engagement ratio. Notably, TikTok videos demonstrated a predominant presence of patient uploaders (29/46, 63%), while YouTube exhibited a higher engagement of health care professionals (31/46, 67.4%). The observed significant difference in viewer engagement and content characteristics between the platforms underlines the need for changing the distribution of health-related content across diverse social media spaces [28] as health care professionals are more likely to use YouTube for their content, which has significantly lower views and engagement ratios.

The variation in video characteristics can also be attributed to the short form of TikTok videos. In particular, educational content was slightly higher on YouTube than on TikTok, which is advantageous in urticaria, a condition that necessitates broader understanding. Specifically, YouTube is designed to hold long-form videos, allowing content for over 1 or 2 hours [35]. In such cases, long-form videos support nuanced comprehension of initiators, stepwise treatment escalation, and chronicity. The step-by-step explanations on YouTube might lead to comprehensive content, reducing common misconceptions. In contrast, the brevity of content on TikTok creates oversimplification of information, limiting discussion of guideline-based handling of urticaria.

### DISCERN, GQS, and ANDI Scores

Our study found that TikTok videos from professionals exhibited significantly higher mean scores across the DISCERN, GQS, and ANDI measures, suggesting a positive association between professional status and the reliability of urticaria-related information. This aligns with the notion that health care professionals bring a level of expertise and accuracy to content creation, potentially enhancing the overall quality of health-related information on social media platforms [36].

Even though condensed and short-form TikTok videos might limit the comprehensive coverage of educational content, online information is likely influenced by content creator attributes. Hill and colleagues [37] note that the quality of TikTok information consumed can be impacted by multiple factors, including content creator characteristics such as their professional medical training, reliance on evidence-based sources, and motivation behind their videos. A similar observation was made by Molitierno et al [38], who noted that videos from health care professionals demonstrated a higher actionability, accuracy, and understandability compared to patient-generated content regarding induction of labor. Consequently, the length of the videos is not the sole determiner of information quality as the characteristics of the creators play a crucial role across social media platforms.

On YouTube, a similar trend was observed, with videos from professional uploaders surpassing those of their nonprofessional counterparts in mean GQS, ANDI, and DISCERN scores. High-quality videos from professionals reinforce the significance of expert-driven communications in diseases such as urticaria [39]. Putting stress on high-quality information among such conditions is vital as management decisions are based on evolving clinical evidence and necessitate individualized assessment [39]. This consistency across platforms underscores the potential influence of the uploader’s background on the quality of urticaria-related content [31]. The higher scores among professional uploaders may be attributed to their expertise, commitment to evidence-based information, and adherence to professional standards [36].

For both platforms, the variations in the quality observed have vital implications for urticaria management. As stated by Zhang et al [40], physicians and patients should be aware of the internet being a source of health-related misinformation even though it is a valuable information source. Similarly, given that chronic urticaria is frequently idiopathic, inaccurate or incomplete information may lead patients to pursue unnecessary trigger elimination strategies, particularly restrictive diets, while delaying escalation to evidence-based therapies such as high-dose antihistamines or biologics. Consequently, it is vital for patients and physicians to critically evaluate YouTube and TikTok information on urticaria as misconceptions might lead to suboptimal treatment, discord between patients and physicians, and delayed diagnosis [41].

This study further explored the impact of various video characteristics on the DISCERN score categories. Notably, nonprofessional uploaders exhibited a higher prevalence of videos in the “poor” and “very poor” categories, emphasizing potential concerns about the quality of the information disseminated by nonprofessionals [42].

Our findings align with existing literature emphasizing the importance of health care professional involvement in health-related content creation. The positive correlation between professional status and content quality is consistent with studies indicating that expert-driven content tends to be more accurate, reliable, and aligned with evidence-based practices [43,44]. The prevalence of verified accounts contributing to higher-quality content corroborates prior research highlighting the role of platform verification in ensuring reliability [43].

### Implications and Recommendations

Urticaria videos on TikTok showed a significantly higher engagement ratio and less accuracy and quality of health-related information than YouTube videos. To enhance the quality of urticaria-related content, collaborations between health care professionals and social media platforms should be encouraged to understand the preferences and expectations of viewers, guiding creators in developing engaging yet accurate health-related content. In addition, initiatives promoting verification and professional engagement can contribute to a more reliable health information environment.

### Limitations

The cross-sectional nature of the analysis provides a snapshot of the current state of urticaria-related content on YouTube and TikTok. Furthermore, the evaluation was based on a specific set of criteria, and the subjective nature of video evaluation may introduce inherent biases. Third, we only included English-language videos in the analysis, which could introduce some limitations. In particular, the findings might not be generalizable to urticaria-related content in populations speaking languages other than English, where the accuracy, quality, or reliability of the information might differ. In addition, our study reflects a cross-sectional evaluation of digital information available at the time of data collection in 2023. Given the rapidly evolving nature of social media platforms such as TikTok and YouTube, the quality and characteristics of available content may have changed since then. However, such snapshot analyses remain important for establishing baseline trends and determining persistent gaps in the quality of online health information. Future studies with larger sample sizes, longitudinal designs, and more diverse evaluation criteria could further refine our comprehension of health-related content on various popular social media platforms and examine how content evolves over time.

### Conclusions

In conclusion, our study offers an exploration of urticaria-related content on YouTube and TikTok, highlighting the influence of uploader types and video characteristics on the perceived quality and accuracy of the information. The findings underscore the importance of health care professional and professional societies’ involvement in content creation and suggest avenues for improving the reliability of health-related information on social media platforms. Involvement in creating accessible, reliable, and evidence-based educational content could enhance the availability of accurate and guideline-based data that may help mitigate the spread of misleading information, support solid health care decisions, and elevate public understanding of urticaria. As these platforms continue to evolve, collaborative efforts among health care professionals, content creators, and platform administrators are essential to cultivate a trustworthy and accurate health information environment.

## Supplementary material

10.2196/88827 Multimedia Appendix 1 Supplementary tools.

## Abbreviations

ANDI: Accuracy in Digital-Health Instrument
GQS: Global Quality Score
IgE: immunoglobulin E
