# Supplementary material for: Accuracy, Reliability, and Quality of YouTube and TikTok Videos as Sources of Information on Urticaria: Cross-Sectional Study
Source: JMIR Dermatol. 2026 Nov 9;9:e88827. doi: 10.2196/88827 (PMC13747268; doi:10.2196/88827)
Supplement: Supplementary file 1 [file derma-v9-e88827-s001.pdf]

Supplementary 1: Tools

Table A. DISCERN instrument for evaluating health information of online videos.

Are the aims clear?	1-5 points
Does the video clip achieve its aims?	1-5 points
Is it relevant?	1-5 points
Is it clear what sources of information were used to compile the video?	1-5 points
Is it clear when the information used or reported in the video was produced?	1-5 points
Is the content balanced and unbiased?	1-5 points
Does the video provide details of additional sources of support and information?	1-5 points
Does it refer to areas of uncertainty?	1-5 points
Does it describe how each treatment works?	1-5 points
Does it describe the benefits of each treatment?	1-5 points
Does it describe the risks of each treatment?	1-5 points
Does it describe what would happen if no treatment was used?	1-5 points
Does it describe how the treatment choices affect overall quality of life?	1-5 points
Is it clear that there may be more than one possible treatment choice?	1-5 points
Does it provide support for shared decision making?	1-5 points
Overall rating of the video based on the answers to all of the above questions?	1-5 points

Table B. Global Quality Score (GQS) used for evaluation of video clips.

Poor quality; poor flow of the video; most information missing; not at all useful for patients	1
Generally poor quality and poor flow; some information listed, but many important topics missing; of very limited use to patients	2
Moderate quality; suboptimal flow; some important information adequately discussed, but other information poorly discussed; somewhat useful for patients	3
Good quality and generally good flow; most of the relevant information listed, but some topics not covered; useful for patients	4
Excellent quality and flow; very useful for patients	5

Table C: Accuracy in Digital-health Instrument (ANDI).

Not accurate at all	0
Minimally accurate	1
Fairly accurate	2
Mostly accurate	3
Completely accurate	4
